# Supplementary material for: Routine Immunisation Coverage Shows Signs of Recovery at Global Level Postpandemic, but Important Declines Persist in About 20% of Countries
Source: Vaccines (Basel). 2025 Apr 3;13(4):388. doi: 10.3390/vaccines13040388 (PMC12031510; doi:10.3390/vaccines13040388)
Supplement: Supplementary file 1 [file vaccines-13-00388-s001.zip › vaccines-3517849-supplementary.pdf]

Title: Routine immunisation coverage shows signs of recovery at global-level post pandemic, but important and declines persist in 20% of countries

Author list: Beth Evans[1*], Laurent Kaiser[2], Olivia Keiser[1], Thibaut Jombart[3]

[1]Institute of Global Health, Faculty of Medicine, University of Geneva, Geneva, Switzerland; [2]Division of Infectious Diseases, Geneva University Hospitals, Geneva, Switzerland; [3]MRC Centre for Global Infectious Disease Analysis, School of Public Health, Imperial College London, UK

Supplementary Materials

1. Further details on methods

A. Coverage trends

Table S1: DTP1 coverage *t*-tests. Global mean expected (ARIMA-modelled) and reported DTP1 coverage from 2020 to 2023, and the calculated difference between the two (delta = expected – reported). 95% confidence intervals (CIs) and associated *p* value from *t*-test.

Year	Expected	Reported	Delta [95% CIs]	p-value
2020	92.8%	91.1%	-1.7% [-1.1%; -2.4%]	< 0.0001
2021	92.7%	90%	-2.7% [-1.7%; -3.8%]	< 0.0001
2022	92.7%	90.9%	-1.8% [-0.7%; -2.8%]	0.001
2023	92.7%	91%	-1.7% [-0.5%; -3.0%]	0.008

Table S2: DTP1 number of immunisations *t*-tests. Number of expected (ARIMA-modelled) and reported (from WUENIC) DTP3 immunisations per year globally from 2020 to 2023, and the calculated difference between the two (delta = expected – reported). 95% confidence intervals (CIs) and associated *p* value reported from *t*-test.

Year	Expected	Reported	Delta [95% CIs]	p-value
2020	623,130	605,628	-17,502 [3,057; -38,060]	0.095
2021	616,587	589,586	-27,001 [-4,434; -49,568]	0.02
2022	612,134	604,708	-7,426 [7,567; -22,419]	0.3
2023	610,452	600,125	-10,327 [13,843; -34,498]	0.4

B. Correlations between predictors

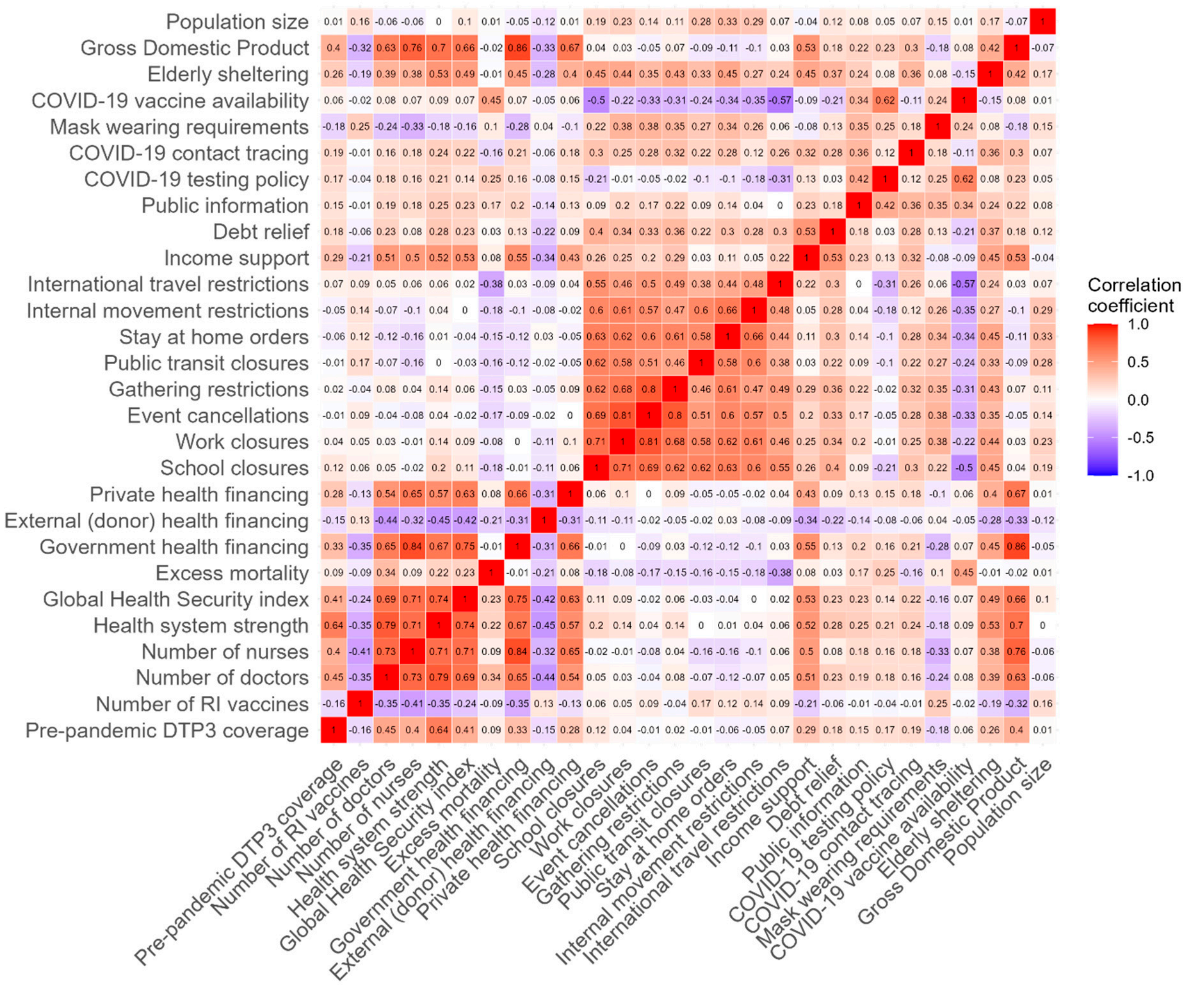

Figure S1: Predictor correlation plot. Pairwise correlation between all combinations of predictors. The less transparent the square, the more correlated two variables are. The numbers in each square indicate the correlation coefficient: -1 indicates perfect negative correlation, 0 means no correlation, and 1 signals perfect positive correlation. Many variables have a coefficient greater than |0.6| indicating strong negative or positive correlation.

2. Further details on results

A. Coverage trends

Table S3: Countries with coverage below expectations in 2023. List of countries with coverage below expectations in 2023 for DTP3. Coverage deltas are reported as reported minus expected coverage, e.g., -5.2% indicates 5.2 percentage points lower than expected. The delta in number of immunisations indicates the number of non-immunised children associated with the coverage delta – i.e., the additional number of missed children not the total number of missed children.

Country	Expected coverage (%)	95% Confidence Interval (low, %)	95% Confidence Interval (high, %)	Reported coverage (%)	Delta (%)	Delta (number immunisations)
India	97.9	91.1	99.0	91.0	-6.9	-1,580,436
Sudan	99.0	83.8	99.0	51.0	-48.0	-782,426
Viet Nam	91.1	71.6	99.0	65.0	-26.1	-357,268
Democratic People's Republic of Korea	97.0	65.6	99.0	16.0	-81.0	-273,506
Mozambique	85.0	73.7	96.3	70.0	-15.0	-181,576
Argentina	92.9	83.1	99.0	66.0	-26.9	-134,437
Uganda	99.0	95.1	99.0	91.0	-8.0	-133,565
Senegal	99.0	84.9	99.0	83.0	-16.0	-83,277
Honduras	92.5	86.0	98.9	73.0	-19.5	-45,039
Ecuador	86.8	77.7	95.8	70.0	-16.8	-44,994
Thailand	97.6	95.6	99.0	92.0	-5.6	-32,901
Benin	75.3	69.6	81.0	69.0	-6.3	-28,847
Paraguay	90.3	84.0	96.6	71.0	-19.3	-26,094
Lebanon	81.9	75.6	88.2	55.0	-26.9	-24,706
Azerbaijan	97.8	90.0	99.0	83.0	-14.8	-18,263
State of Palestine	99.0	92.5	99.0	88.0	-11.0	-15,893
Panama	88.0	67.9	99.0	66.0	-22.0	-15,550
Kyrgyzstan	96.1	92.0	99.0	86.0	-10.1	-15,046
Spain	96.5	94.6	98.4	93.0	-3.5	-11,764
Sweden	98.0	96.0	99.0	94.0	-4.0	-3,911
Czechia	97.5	95.7	99.0	94.0	-3.5	-3,221
Vanuatu	94.0	84.6	99.0	72.0	-22.0	-1,952

Country	Expected coverage (%)	95% Confidence Interval (low, %)	95% Confidence Interval (high, %)	Reported coverage (%)	Delta (%)	Delta (number immunisations)
North Macedonia	94.4	90.4	98.4	86.0	-8.4	-1,412
Switzerland	96.6	95.2	98.1	95.0	-1.6	-1,362
Slovenia	94.6	91.3	97.9	89.0	-5.6	-999
Lithuania	94.3	91.3	97.2	90.0	-4.3	-911
Belize	95.0	89.6	99.0	85.0	-10.0	-735
Albania	99.0	97.5	99.0	97.0	-2.0	-572
Cyprus	97.4	95.3	99.0	95.0	-2.4	-347
Dominica	97.2	93.1	99.0	56.0	-41.2	-297
Grenada	95.5	88.5	99.0	86.0	-9.5	-130
Saint Vincent and the Grenadines	98.3	96.2	99.0	94.0	-4.3	-52

B. Discriminant Analysis of Principal Components

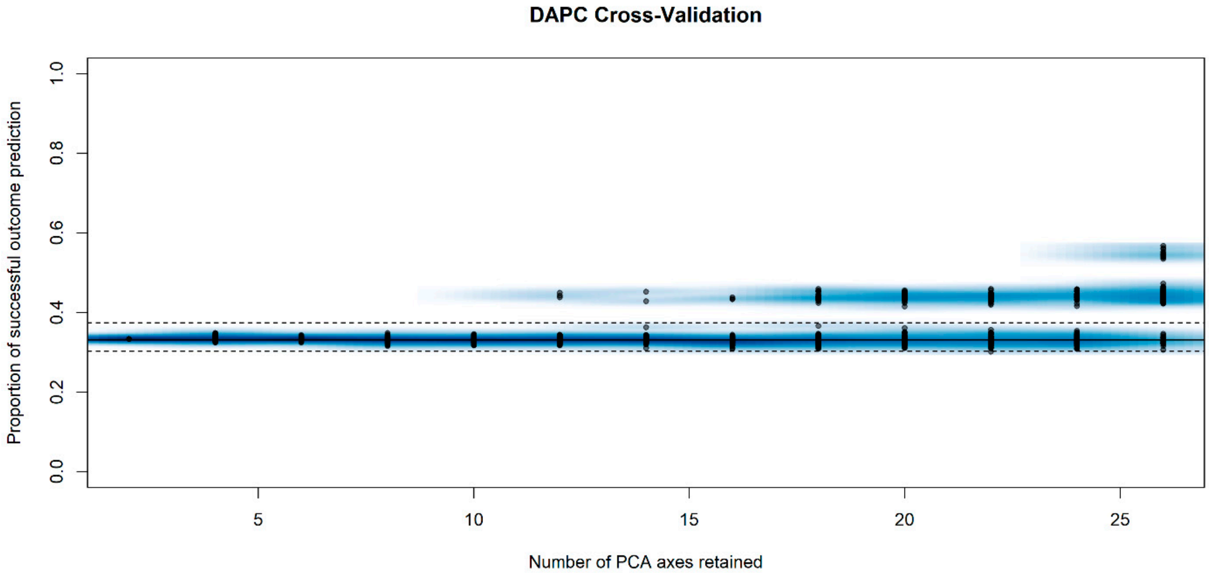

Figure S2: Cross-validation of Discriminant Analysis of Principal Components. Cross validation plot for classifying country performance into below expectations, within expectations, and above expectations. The cross validation is conducted on the test (30%) set of data points, with 100 repetitions per number of Principal Component Axes retained (x-axis). The y-axis shows the proportion of successful outcomes per run across the test dataset, i.e., the proportion of data points that are correctly classified. The horizontal dotted lines indicate classification if by chance, and dots further above these horizontal lines indicate more successful classification. This plot

shows that below 12 PCAs the model was not better than chance at classifying country performance. Above this level, performance increases slightly as more PCAs are retained.

Table S4: Discriminant Analysis of Principal Components confusion matrix. Data covering all 616 countries. The rows describe the actual classes to which the countries being classified belong, and the columns describe the classes to which the model predicts the classes below. The class error reports the percentage of time the country is misclassified.

		**Predicted**			**Class error**
		Below expectations	Within expectations	Above expectations	
**Actual**	Below expectations	2	111	1	98.2%
	Within expectations	8	481	4	2.4%
	Above expectations	0	4	5	44.4%

C. Random Forest

Table S5: Random Forest confusion matrix for the train dataset. Train data includes 70% of data (431 countries). The rows describe the actual classes to which the countries being classified belong, and the columns describe the classes to which the model classifies the countries based on the Random Forest. The class error reports the percentage of time the country is misclassified.

		**Predicted**			**Class error**
		Below expectations	Within expectations	Above expectations	
**Actual**	Below expectations	13	71	0	84.5%
	Within expectations	13	328	0	3.8%
	Above expectations	0	6	0	100%

Table S6: Random Forest confusion matrix for the test dataset. Test data includes 30% of data (185 countries). The rows describe the actual classes to which the countries being classified belong, and the columns describe the classes to which the model classifies the countries based on the Random Forest. The class error reports the percentage of time the country is misclassified.

		**Predicted**			**Class error**
		Below expectations	Within expectations	Above expectations	
	Below expectations	6	3	0	33.3%

**Actual**	Within expectations	24	149	3	15.3%
	Above expectations	0	0	0	NA

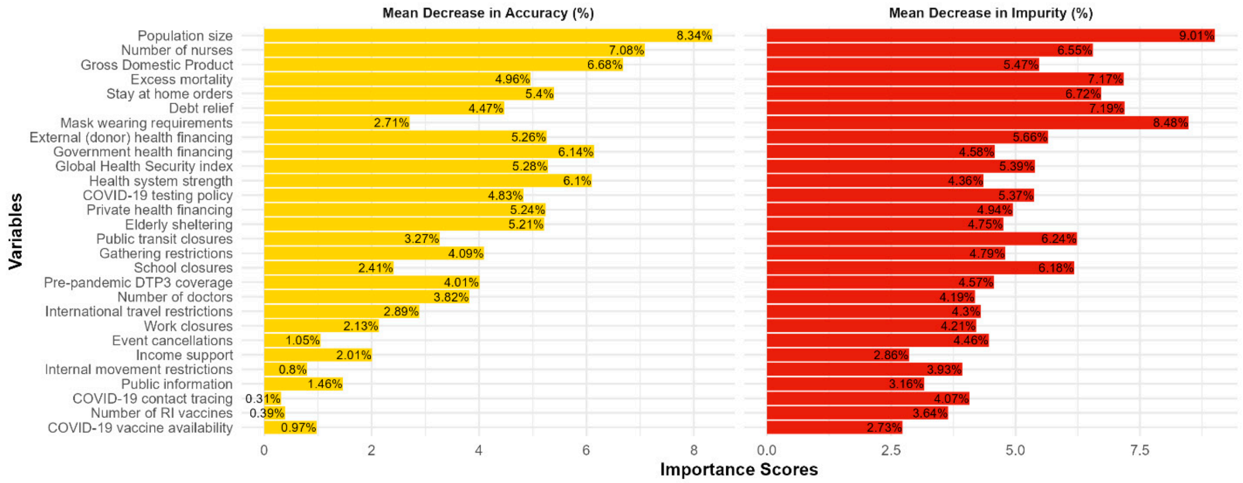

Figure S3: Summary of relative predictive importance of each predictor variable when investigating non-linear explanatory power through constructing a Random Forest. The mean decrease in accuracy quantifies the decrease in model accuracy when each predictor is excluded individually. The mean decrease in impurity, also known as the Gini index, calculates the reduction purity of nodes and splits within the trees of the Random Forest when each variable is excluded. Variables are ordered by the sum of both importance scores from largest to smallest.
